# Improved topical delivery of curcumin by mussel adhesive protein functionalized ethosomes for effective psoriasis treatment

**DOI:** 10.1016/j.ijpx.2026.100600

**Published:** 2026-07-04

**Authors:** Jiangxiu Niu, Ming Yuan, Liye Wang, Pei Zhang, Jucai Wang, Xianming Liu

**Affiliations:** a College of Food and Drug, Luoyang Normal University, Luoyang, Henan 471934, PR China; b College of Life Science, Luoyang Normal University, Luoyang, Henan 471934, PR China

**Keywords:** Mussel adhesive protein, Ethosomes, Curcumin, Topical delivery, Anti-psoriatic efficacy

## Abstract

Psoriasis is a prevalent inflammatory skin disorder exhibiting a rapidly increasing incidence. Curcumin (Cur) serves as an effective therapeutic agent for psoriasis and is commonly administered through the cutaneous route. Nevertheless, the poor skin permeability and retention of Cur restrict its therapeutic efficacy against psoriasis. In this study, we fabricated mussel adhesion protein (MAP)-modified Cur-loaded ethosomes (Cur-MAP-Es) aimed at enhancing both the permeation and retention of Cur within the skin for improved topical treatment of psoriasis. The average particle size of Cur-MAP-Es was 197.17 nm, and the encapsulation efficiency was 90.84%. The Cur-MAP-Es exhibited a spherical morphology, along with high elasticity, favorable stability, and a prolonged release pattern within 24 h. Additionally, the Cur-MAP-Es exhibited a 3.47-fold higher skin retention compared to the Cur—Es. Intradermal fluorescence distribution analysis indicated that most of the Cur in the Cur-MAP-Es was effectively retained in the epidermis after being delivered into the skin via vesicles. The interaction mechanisms of Cur-MAP-Es with the skin have revealed that Cur-MAP-Es can weaken the skin barrier, thereby facilitating enhanced permeability and drug retention. Furthermore, Cur-MAP-Es could significantly alleviate the inflammation in the mouse model of psoriasis. These results suggest that Cur-MAP-Es may serve as an effective strategy to enhance the topical delivery efficiency of Cur, thereby showing considerable potential in the management of psoriasis.

## Graphical abstract

**Figure:**
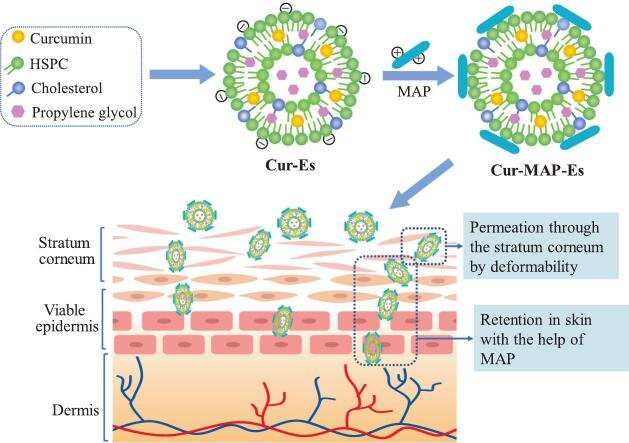

## 1 Introduction

Psoriasis is a chronic inflammatory skin disease characterized by abnormal keratinocyte proliferation and sustained inflammatory cell infiltration(Kishta et al., 2025). Topical administration is widely used in the clinical management of psoriasis, as it enables drugs to act directly on inflammatory sites and provide targeted control of disease symptoms. Moreover, compared with systemic administration pathways, topical application of drugs can decrease the systemic absorption, thereby lowering the risk of systemic adverse reactions. However, the stratum corneum (SC) restricts the penetration of drugs across the dermal barrier, leading to compromised drug bioavailability and limited clinical effectiveness when applied topically.

Accumulating clinical studies have demonstrated that curcumin(Cur) can effectively alleviate clinical symptoms and improve skin lesions in patients with psoriasis, confirming its reliable clinical application value in the treatment of this disease(Kantasa et al., 2025; Mo et al., 2024). However, its instability, low water solubility, and insufficient cutaneous permeation and retention severely limit the therapeutic application of Cur(Liston et al., 2025). Additionally, the SC barrier composed of keratinocytes and intercellular lipids also poses significant challenges to the percutaneous absorption of Cur. Consequently, an advanced delivery system is needed for Cur to facilitate deeper skin penetration and prolonged site retention for achieving superior topical therapeutic efficacy.

To address the poor absorption of Cur in the skin, topical administration using nanoparticles have gained increasing importance(Aydin et al., 2024). Up to now, various nanoparticles have been developed for localized delivery of Cur, including solid lipid nanoparticles, nanogels, nanoemulsions and nanocrystals(Aydin et al., 2024; Khatoon et al., 2021; Niu et al., 2022a; Xiang et al., 2023). The active development and implementation of these topical drug delivery systems have effectively resolved numerous issues related to skin permeation as well as absorption and bioavailability. Liposomes are closed, nano-sized vesicles constructed from phospholipid bilayers. They exhibit excellent biocompatibility and cellular affinity, and can encapsulate lipophilic, hydrophilic, and amphiphilic drugs. By enhancing the hydration of the SC, traditional liposomes can promote the permeation of entrapped drugs into the skin through diffusion and capillarity, thus improving cutaneous drug absorption(Carvalho et al., 2025). However, conventional liposomes demonstrate limited permeation into deeper skin layers owing to their rigid bilayer structure. The incorporation of edge-active agents allows for significant improvement in the elasticity and deformability of liposomes(Jahan et al., 2025). However, the inclusion of edge activators could compromise the biocompatible nature of liposomes. Ethosomes are a novel type of deformable nanocarrier derived from liposomal systems, formulated by incorporating 20–45% biocompatible short-chain alcohols into phospholipid bilayers(Paiva-Santos et al., 2021). The introduction of these short-chain alcohols can enhance the fluidity of lipid bilayers and improve the entrapment efficiency of lipophilic drugs(Waghule et al., 2020). However, conventional nanoparticles used for topical drug delivery, including ethosomes, often struggle to effectively retain in skin for local treatment. To overcome this limitation, the use of surface-modified nanocarriers has become an effective strategy to optimize the topical delivery efficiency of Cur. Previous studies have reported hyaluronan-modified ethosomes and MAP-modified polymer micelles(Niu et al., 2025; Zhang et al., 2019). However, hyaluronan is mainly mediated by the CD44 receptor, and its biological adhesion to the skin tissue is relatively weak, while polymer micelles lack effective penetration ability through the SC. Recently, it has been proven that cationic ethosomes can remarkably enhance the skin targeting behavior of the drug(Vishwakarma et al., 2025).

Mussel adhesion protein (MAP) is a type of protein secreted by mussels that has garnered considerable interest as a promising adhesive material for biomedical applications, owing to its excellent adhesion properties along with favorable biocompatibility(Yan et al., 2026). MAP is abundant in 3, 4-dihydroxyphenylalanine (DOPA), and its catechol-mediated chelation and hydrogen bonding interactions with the substrate are considered to be the foundation of its biological adhesion(Valappil et al., 2025). Additionally, the molecular architecture of MAP is abundant in cationic amine residues, such as lysine, arginine, and tyrosine, which can enhance the affinity with negatively charged cell membranes(Xu et al., 2025). In the field of nanomedicine, MAP has been used to develop nanoparticles designed to increase the retention of drugs within the lesion, thus boosting the therapeutic outcomes and mitigating the untoward effects of the drugs(Jeong et al., 2018). In view of this, MAP might be used in the design of topically applied carriers to improve the affinity between the carrier and the skin by taking advantage of the biological adhesion and cationic properties of MAP, thereby improving the skin retention and local efficacy of drugs.

In this study, we aim to construct MAP-modified ethosomes (Cur-MAP-Es) as potential nanocarrier to enhance the skin delivery of Cur. We expect that Cur-MAP-Es will combine the excellent skin permeability of ethosomes with the good tissue adhesion of MAP, which can not only effectively break through the SC barrier, but also realize the effective retention of drugs in skin lesions, thereby producing better topical therapeutic effects for psoriasis. Therefore, Cur-MAP-Es was formulated and its physicochemical properties were evaluated. The ability of Cur-MAP-Es to promote the cutaneous delivery of drugs was verified through the in vitro skin permeation and retention assays. We also investigated the interaction mechanisms between Cur-MAP-Es and the skin. In addition, the therapeutic effectiveness of Cur-MAP-Es was assessed in psoriatic-like mouse models. The enhanced topical delivery effect of Cur-MAP-Es effectively alleviated skin lesions in psoriasis model mice, demonstrating its application potential in psoriasis therapy.

## 2 Materials and methods

### 2.1 Materials

Curcumin (purity ≥98%) was supplied by Ivy Biotechnology Co. Ltd. (Xian, China). Hydrogenated soybean phospholipid (HSPC), Cholesterol and propylene glycol were acquired from McLean Biochemical Technology Co., Ltd. (Shanghai, China). Mussel adhesive protein (MAP, molecular weight 10 kDa) was procured from Jiangyin Bairui Biochemical Technology Co., Ltd. (Jiangyin, China). Hematoxylin and eosin (HE) and 4% paraformaldehyde were purchased from Meilun Biotechnology Co., Ltd. (Dalian, China). Skin of Bama miniature pigs aged one month was obtained from Linxi County Jingde Agricultural Products Sales Co., Ltd. (Hebei, China). Imiquimod cream (5%) was purchased from Minxin Pharmaceutical Co., Ltd. (Chengdu, China). Clobetasol propionate was purchased from Jiangsu Zhiyuan Pharmaceutical Co., Ltd. (Wuxi, China). All other chemical reagents were of analytical purity.

### 2.2 Preparation of Cur-MAP-Es

Cur was encapsulated into ethosomes by using the pH gradient method(Abu-Huwaij and Zidan, 2024; Zhang et al., 2016). In brief, a mixture of HSPC (60 mg), cholesterol (30 mg), and Cur (6 mg) was prepared in 4 mL of propylene glycol, the mixture was heated and dissolved at 60 °C. Subsequently, the pH of the system was regulated to 8.5 via the gradual addition of aqueous ammonia. The physiological saline (16 mL) was then slowly added dropwise with stirring at 750 rpm prior to dark incubation at 30 °C for 1.5 h. During the neutralization stage, acetic acid solution (1 mol/L) was incorporated into the system to adjust the pH to 7.0. Unionized Cur was capable of traversing the lipid bilayer and being entrapped in the vesicles, thereby generating homogeneous Cur-loaded ethosomes (Cur—Es). MAP aqueous solution was then slowly added to Cur-Es under constant stirring, and the mixture was then maintained at 37 °C under continuous orbital agitation for 3 h to achieve the MAP-functionalized Cur-Es (Cur-MAP-Es). Due to the negative zeta potential of Cur—Es, MAP was easily adsorbed on the surface of the vesicles.

### 2.3 Characterization of Cur-MAP-Es

#### 2.3.1 Encapsulation efficiency (EE)

The EE of the formulated Cur-MAP-Es was evaluated using an ultrafiltration-centrifugation technique(Niu et al., 2022b). Briefly, 0.4 mL of Cur-MAP-Es dispersion was transferred to a 30 *k*D ultrafiltration tube (Milipore Co. Ltd., USA) and centrifuged at 3000 rpm for 30 min. The acquired filtrate was employed to determine the amount of unencapsulated Cur, the concentration of which was measured via UV–Vis spectroscopy (TU-1810PC, Purkinje, China) at 425 nm. The EE percentage was then computed based on the equation below:

$$\mathrm{EE}\%=\left[\frac{\text{Total}\;\mathrm{Cur}–\text{Unencapsulated}\;\mathrm{Cur}}{\text{Total}\;\mathrm{Cur}}\right]\times 100$$

#### 2.3.2 Particle size, zeta potential and surface morphology

The vesicle size, size distribution and zeta potential of Cur-Es and Cur-MAP-Es were characterized using a Zetasizer (ZS90, Malvern, UK) based on dynamic light scattering (DLS). DLS tests were conducted at 25 °C with a fixed 90° scattering angle, and the suspension was diluted to proper concentration using deionized water prior to detection. The surface morphology of Cur-Es and Cur-MAP-Es was characterized via scanning electron microscopy (SEM, Sigma 500, ZEISS, Germany). The diluted suspension was dropped onto silicon substrates and air-dried, followed by vacuum gold sputtering before SEM observation at an accelerating voltage of 3–5 kV.

#### 2.3.3 Elasticity determination

The elasticity of Cur-Es and Cur-MAP-Es was measured by membrane filtration method(Hussain et al., 2023). Briefly, samples were passed through 0.22 μm polycarbonate filters at a constant pressure of 0.45 MPa. The volume of dispersion permeated within 5 min was recorded, and the particle size was determined before and after extrusion using the Zetasizer. Elasticity was derived from the equation:

$$E=J_{\mathrm{flu}}\times {\left(\frac{\mathrm{Rv}}{\mathrm{Rp}}\right)}^{2}$$

Where E represents the vesicle elasticity, J is defined as the volume of ethosome dispersion extruded in 5 min, with Rv and Rp representing the vesicle sizes after and before extrusion, respectively.

#### 2.3.4 Stability study

The short-term stability of Cur-Es and Cur-MAP-Es was assessed to investigate whether vesicle aggregation or drug leakage occurred during storage. Formulations were aliquoted into glass vials and protected from light at 4 °C. At designated intervals (days 0, 1, 3, and 7), samples were taken to evaluate the physicochemical properties.

### 2.4 In vitro drug release

The assessment of drug release from Cur solution, Cur-Es and Cur-MAP-Es was conducted using the dialysis method(Ilyas et al., 2024). Briefly, the Cur formulations were respectively introduced into the cellophane membrane dialysis bag (8–14 kDa, MWCO), which were then sealed at both ends and checked for leakage. The dialysis bags were soaked in 40 mL PBS (pH 7.4) containing 1% Tween-80 and incubated at 37 °C with 50 rpm agitation. At set intervals, 2.0 mL aliquots of release medium were collected and replaced with an equal volume of fresh pre-warmed medium. Cur concentration was determined by UV–Vis spectroscopy.

### 2.5 Fourier transform infrared (FTIR) spectroscopy

FTIR spectra of Cur, physical mixture of Cur and blank MAP-Es, blank MAP-Es, and Cur-MAP-Es were separately recorded in the range of 4000–400 cm^−1^ at a resolution of 4 cm^−1^ at ambient temperature(Nicolet iS5, Thermo, USA). Each sample measurement was replicated three times to ensure consistency in the resulting spectra. This replication process aimed to evaluate the accuracy of transmittance measurements, which might be influenced by sample uniformity.

### 2.6 In vitro assessment of skin permeation and retention

#### 2.6.1 In vitro skin permeation

Skin permeability measurements of Cur solution, Cur—Es, and Cur-MAP-Es were performed using Franz-type diffusion cells(RYJ-6B, Huanghai, China)(Fang et al., 2024). Bama miniature porcine skin was mounted between donor and receptor compartments, with SC oriented toward the donor side. The diffusion device offered an available permeation surface of 2.8 cm^2^ toward the skin. The receptor compartment was filled with 6.5 mL PBS (pH 7.4) containing 1% Tween-80 and continuously stirred at 300 rpm. The receptor compartment was thermostatically controlled at 37 °C by means of a water circulation bath. A 0.5 mL aliquot of each Cur formulation, containing 0.2 mg of the drug, was administered to the donor compartment. At fixed time points, 1 mL samples were taken and instantly replaced with pre-warmed medium. The duration of permeation test was 12 h. Cur content was determined by high-performance liquid chromatography (HPLC) (U-3000, Thermo, USA) using a Wondersil C18 column (5 μm, 250 × 4.6 mm). Separation was performed with a mobile phase of acetonitrile and 0.5% phosphoric acid (58:42, *v*/v) at 1.0 mL/min, with the column temperature set at 30 °C. The detection wavelength was 425 nm, and the injection volume was 20 μL. The cumulative drug permeation per unit area (Q_n_) corresponding to every time point was derived through calculations based on the equation:

$$Q_{n}=\frac{V_{0}C_{n}+V_{i}\sum_{i=0}^{n-1}C_{i}}{A}$$

where V_0_ is the total volume of the receptor compartment, C_n_ is the drug concentration at the nth sampling time point, V_i_ and C_i_ are the sampling volume and corresponding drug concentration, respectively, the subscript *i* = 0 denotes the initial sampling point at time zero prior to the start of the permeation experiment, and A represents the effective diffusion area of the device.

#### 2.6.2 In vitro skin retention

Following the permeation experiment, the skin from the diffusion device was gently harvested(Zhao et al., *2023*). The skin was fully wiped with ethanol and then washed with deionized water to ensure complete removal of surface drug residues. Subsequently, the treated skin was dried *using filter paper and cut into small fragments.* These fragments were immersed in 2 mL of chromatographic methanol and refrigerated at 4 °C for 24 h, after which intermittent ultrasound was applied for 30 min to assist in the extraction process*.* The sample was centrifuged at 3000 rpm for 15 min, and the supernatant was filtered before HPLC analysis. Finally, the skin retention of Cur was calculated.

### 2.7 Intradermal fluorescence distribution study

To visually observe the penetration depth and intradermal distribution of Cur, 0.5 mL aliquots of Cur solution, Cur—Es, and Cur-MAP-Es with equal Cur concentrations were separately loaded onto porcine skin in Franz diffusion cells. Following collection of skin samples at designated time points (2 h and 8 h), residual surface formulation was eliminated by rinsing with deionized water. Three samples from the treated skin were vertically sectioned into slices measuring 10 μm in thickness using a frozen-section microtome (CM1950, Leica, Germany). The skin slices were stained with DAPI and subsequently imaged with an automated slide scanning system (3DHistech, Budapest, Hungary).

### 2.8 Interaction mechanisms of Cur-MAP-Es with the skin

#### 2.8.1 HE analysis of skin structure

After separate treatment with Cur solution, Cur—Es, and Cur-MAP-Es for 12 h following the same protocol as the in vitro skin permeation study, porcine skin samples were collected. Porcine skin without any treatment served as the control. The harvested skin samples were fixed in 4% paraformaldehyde, followed by dehydration in a graded ethanol series, xylene clearing, and paraffin embedding. The paraffin-embedded tissues were cut into 5 μm serial sections, stained with hematoxylin and eosin (HE), air-dried, and mounted with neutral balsam. Finally, the morphological alterations in skin microstructure induced by the different formulations were visualized with a 3DHistech system.

#### 2.8.2 SEM of skin surface

After 12 h of separate treatment with Cur solution, Cur—Es, and Cur-MAP-Es, porcine skin specimens were analyzed via SEM to observe treatment-induced surface microstructural alterations. Untreated porcine skin was used as the control. The skin samples were rinsed with deionized water, followed by immersion in 4% paraformaldehyde to achieve structural preservation via fixation. Following thorough dehydration by lyophilization, the skin samples were subjected to gold sputter-coating to enhance electrical conductivity prior to SEM imaging.

#### 2.8.3 DSC analysis of skin thermal behavior

Following separate 12 h treatment with Cur solution, Cur—Es, and Cur-MAP-Es, porcine skin samples were cleaned with deionized water, freeze-dried, and sectioned into small fragments. Untreated porcine skin served as the control. About 6 mg of the sample fragments were transferred into an aluminum pan and subsequently placed into the furnace chamber of a differential scanning calorimeter (DSC, Q2000, TA, USA) for separate analysis. A scanning rate of 10 °C/min was applied during thermal analysis, with measurements taken from 0 to 200 °C. DSC curves were obtained from skin samples with and without prior treatment to investigate the alterations in thermal behavior induced by various Cur formulations.

#### 2.8.4 FTIR analysis of skin lipid and keratin

After 12 h of separate treatment with Cur solution, Cur—Es, and Cur-MAP-Es, the skin was removed from the Franz diffusion cells, rinsed with deionized water, freeze-dried, and cut into small pieces. Untreated porcine skin served as the control. The skin samples were then subjected to FTIR analysis for spectral data collection. Spectra were recorded in the range of 400–4000 cm^−1^, with a spectral resolution of 4 cm^−1^ and 64 cumulative scans. Spectral analysis was performed to characterize the bond vibrations and stretching modes related to key skin components, including skin proteins, carbohydrates, and lipids (Hussain et al., 2024).

#### 2.8.5 TEWL measurements

The initial transepidermal water loss (TEWL) values of porcine skin were detected by an evaporimeter (Tewameter® TM 300, Courage & Khazaka, Germany). Cur solution, Cur—Es, Cur-MAP-Es, and physiological saline (control) were separately applied to porcine skin for 12 h. After treatment, the application site was rinsed with physiological saline and blotted dry. Following stabilization of the perpendicularly positioned TEWL probe within 60 s, measurements were recorded at baseline (0 h) and 24 h post-treatment, with results expressed in g/(h·m^2^) and compared to the control group.(Pretel-Lara et al., 2024).

### 2.9 The ameliorative effect on psoriasis-like lesions

Female KM mice (18–22 g) were randomly allocated into six groups: normal, model, clobetasol propionate (CP), Cur solution, Cur-Es and Cur-MAP-Es, each group contained 6 mice. All groups except the normal control received a daily topical application of imiquimod (IMQ) cream (25 mg/cm^2^) on the right ear for 7 successive days to induce psoriasis-like dermatitis. In the treatment phase, mice excluding those in the normal and model groups were treated once daily with 0.2 mL/cm^2^ of their respective Cur formulation on the right ear, while the CP group received 45 mg/cm^2^ of CP ointment daily(Takezaki et al., 2023). Skin inflammation in mouse ears was evaluated every other day according to the Psoriasis Area and Severity Index (PASI), where erythema, desquamation, and thickening were individually scored from 0 (absent) to 4 (severe). The total score (0−12) quantified overall disease severity. On day 8 post-treatment, animals were sacrificed, and ear samples were harvested for histological processing. Digital scanning of the prepared sections was carried out using a 3DHistech system.

### 2.10 Statistical analysis

All experiments were conducted in triplicate, and data are presented as mean ± SD. Statistical evaluation was performed using Student's *t*-test for two-group comparisons and one-way ANOVA for comparisons among three or more groups. Differences were considered statistically significant at *p < 0.05, **p < 0.01, and ***p < 0.001.

## 3 Results and discussion

### 3.1 Optimization of the Cur-MAP-Es formulation

Cur-Es exhibited a negative zeta potential, allowing positively charged MAP to be easily adsorbed onto the surface of the vesicles. In the process of formulating Cur-MAP-Es, HSPC was selected to form phospholipid membrane, which could help prevent oxidation and enhance the stability of the formulation. Ethanol is commonly employed in the formulation of ethosomes, but it has notable drawbacks such as volatility and irritation to damaged skin(Zhang et al., 2019). In this study, propylene glycol was chosen as an alternative for formulating ethosomes. Propylene glycol is non-volatile, mild, and non-irritating to the skin while also providing moisturizing properties(Kang et al., 2022). It is commonly used as a moisturizing agent in both transdermal drug delivery systems and cosmetic products. Previous research indicated that ethosomes formulated with propylene glycol demonstrated superior skin retention compared to traditional liposomes and ethanol-formulated ethosomes(Zhang et al., 2023). During formulation development, it was found that when the propylene glycol concentration exceeded 25%, there was a significant increase in particle size of the vesicles, while when its concentration fell below 15%, there was a marked decrease in drug encapsulation efficiency. Therefore, an optimal concentration of propylene glycol was determined to be 20%.

In addition, the influence of MAP concentration on the formulation of Cur-MAP-Es was also evaluated. Ideally, an increase in the amount of MAP on the vesicle surface could enhance the skin retention capacity of the formulation. However, excessive MAP within the formulation might lead to system instability. The impact of varying MAP concentrations (0, 0.5, 1.0, 2.0, 3.0 and 4.0 mg/mL) on the formulation was assessed. As shown in Table 1, with increasing MAP concentration, the vesicle particle size gradually increased, whereas the zeta potential decreased as a result of electrostatic adsorption. Upon increasing the MAP concentration to 4 mg/mL, the particle size reached 385.49 nm and EE reduced to 71.90%, accompanied by a broad particle size distribution. This phenomenon might be due to the excess of MAP adsorbed onto the vesicle surface which disrupted the hydrophobic inner structure and led to significant precipitation of Cur(Sonali et al., 2016). Compared with vesicles without MAP adsorption, MAP at concentrations of 2.0 and 3.0 mg/mL increased particle sizes by approximately 10 nm and 20 nm, respectively. No significant differences were observed in particle size, zeta potential or encapsulation efficiency between the two groups. Considering that a higher MAP concentration may better improve skin retention, 3.0 mg/mL was ultimately chosen as the optimal concentration.

**Table 1:** Influence of varying MAP concentrations on Cur-MAP-Es.

Concentration of MAP (mg/mL)	Particle size (nm)	PDI	Zeta potential (mV)	EE (%)
0	177.04 ± 2.19	0.18 ± 0.06	−36.28 ± 3.16	94.17 ± 0.54
0.5	179.83 ± 1.16	0.19 ± 0.04	−36.03 ± 1.76	97.61 ± 0.43
1	182.87 ± 0.87	0.20 ± 0.02	−30.97 ± 1.20	95.06 ± 0.63
2	189.65 ± 1.36	0.21 ± 0.03	−27.36 ± 3.85	94.24 ± 0.23
3	197.17 ± 2.44	0.26 ± 0.01	−24.14 ± 1.86	90.84 ± 0.42
4	385.49 ± 4.62	0.45 ± 0.15	−17.83 ± 3.92	71.95 ± 0.34

### 3.2 Characterization of Cur-MAP-Es

#### 3.2.1 Drug incorporation and EE

**Fig. 1:**
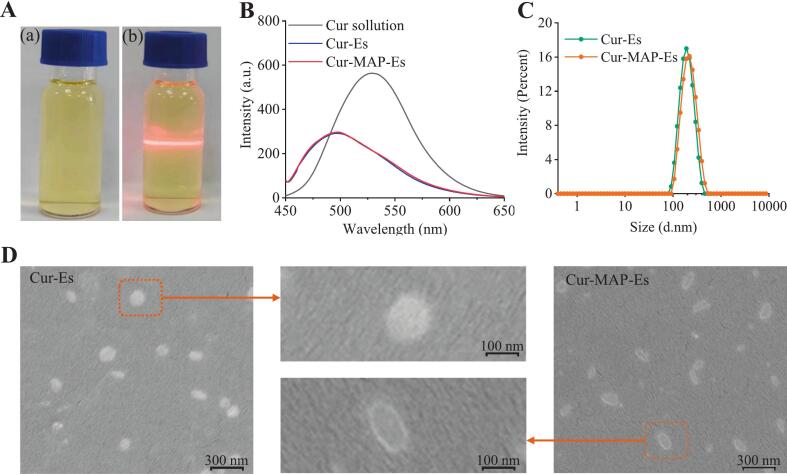
Characterization of Cur-MAP-Es. (A) Appearance and Tyndall phenomenon caused by light scattering when the light beam passed through the formulation. (B) Fluorescence spectral profiles of Cur-solution, Cur-Es and Cur-MAP-Es. (C) Size distribution of Cur-Es and Cur-MAP-Es. (D) SEM micrographs of Cur-Es and Cur-MAP-Es.

The developed Cur-MAP-Es formed a yellow and transparent liquid, which exhibited an obvious Tyndall phenomenon when irradiated with laser light (Fig. 1A). This phenomenon was attributed to the scattering of light by colloidal particles, indicating that Cur-MAP-Es formed a colloidal dispersion. In the fluorescence spectrum, both Cur-Es and Cur-MAP-Es displayed a Cur emission peak at 495 nm, whereas the peak for the Cur solution was observed at 533 nm (Fig. 1B). Notably, the fluorescence emission band of the encapsulated drug demonstrated an evident redshift. Furthermore, the fluorescence intensity of the encapsulated drug was significantly reduced compared to that of an equivalent concentration of Cur solution. This reduction could be ascribed to self-quenching behavior resulting from increased local concentrations of Cur, thereby confirming successful encapsulation of Cur in the vesicles. The EE serves as a pivotal indicator for assessing the performance of a drug delivery system, being crucial for achieving successful therapeutic outcomes(Wang et al., 2024). The EE of Cur-Es and Cur-MAP-Es was found to be 94.17 ± 0.54% and 90.84 ± 0.42%, respectively, indicating notable affinity and compatibility in the drug-carrier interactions, and MAP modification showed little influence on the encapsulation of Cur. However, it was noted that a modest reduction in the EE of Cur-MAP-Es was observed relative to that of Cur—Es. The incorporation of MAP seemed to result in a reduction in drug EE, which might be due to the insertion of hydrophobic chains from MAP into the phospholipid bilayers, compromising the vesicle integrity and leading to minor leakage of entrapped drug(Cai et al., 2021). The Cur concentration of Cur-Es and Cur-MAP-Es was 0.23 ± 0.15 mg/mL and 0.21 ± 0.17 mg/mL, respectively. In conclusion, the Cur-MAP-Es could encapsulate substantial doses of Cur and is anticipated to improve the topical therapeutic effect of Cur.

#### 3.2.2 Particle size and zeta potential

Particle size and zeta potential are critical factors affecting the skin permeability and stability of nanoparticles. By DLS measurement, the average particle size of Cur-Es and Cur-MAP-Es was determined to be 177.04 nm and 197.17 nm, with corresponding PDI values of 0.18 and 0.26, respectively. A smaller particle size is particularly crucial for transdermal drug delivery, as nano-sized particles could easily overcome the SC barrier, which is the primary obstacle to skin drug delivery(Khan et al., 2025). Furthermore, these nanoparticles are capable of enhancing the permeability of hydrophobic drugs that generally exhibit weak percutaneous permeation. It is worth noting that nanoparticles modified with MAP exhibited a larger particle size compared with unmodified nanoparticles, and this size discrepancy might be due to the MAP coating applied to the modified particles. The size distributions for Cur-Es and Cur-MAP-Es are presented in Fig. 1C, and both formulations exhibited narrow particle size distributions with a PDI below 0.3, indicating that the developed nanoparticles were mono-disperse and the distribution of particles in the formulation was uniform, which was useful for transdermal drug delivery systems of topical therapy. We also evaluated the zeta potential of Cur-Es and Cur-MAP-Es. Zeta potential is indicative of nanoparticle stability in the system. In general, an absolute zeta potential greater than 20 mV implies stronger electrostatic repulsion between nanoparticles, leading to enhanced stability properties to the formulation(Kim et al., 2024). Cur-Es and Cur-MAP-Es displayed zeta potentials of −36.28 mV and − 24.14 mV, respectively. The decrease in zeta potential following MAP coating suggests that the successful association of MAP with the particle surface altered the surface charge characteristics of the particles(Dong et al., 2015). Negative surface charge is beneficial to promote drug retention in skin due to the anionic nature of skin cell membrane(Zhang et al., 2020).

#### 3.2.3 Surface morphology

Fig. 1D presented the SEM micrographs of the vesicles, which revealed that they were nearly spherical in shape, exhibiting smooth surface with no aggregation. Cur-MAP-Es presented a typical shell-like morphology, with a dark central region and a bright peripheral surface. This might be due to the moisture retention properties of MAP molecules on the surface of the vesicles, resulting in water entrapped in MAP did not fully evaporate during the drying process. In addition, scale bar estimation from SEM micrographs suggested a diameter of approximately 140 nm for Cur-MAP-Es. The particle size observed by SEM was smaller than the hydrodynamic diameter measured by DLS, which could be explained by the fact that SEM determines the geometric size of dry particles in a vacuum, whereas DLS determines the hydrodynamic diameter of hydrated particles together with their solvation layers in aqueous solution(Aghaz et al., 2023).

#### 3.2.4 Elasticity

The elasticity of the vesicle carrier is a critical factor in facilitating skin permeation of drugs(Esposito et al., 2022). Propylene glycol has been shown to reduce the stiffness of the lipid bilayer, thereby enhancing the flexibility or elasticity of the vesicles, allowing them to morph into smaller sizes and adapting their form by responding locally to ambient tension(Iizhar et al., 2016). The measured elasticity values of Cur liposomes, Cur—Es, and Cur-MAP-Es were 4.28 ± 0.37, 19.20 ± 0.58 and 18.49 ± 0.63, respectively. The observed values revealed enhanced elasticity in the ethosomal systems compared to liposomal formulations, a property ascribed to the flexibility provided by propylene glycol. This component exerted an effective influence on the lipid bilayers, imparted structural flexibility to ethosomes without inducing any rupture, and could act as a promising delivery system to improve skin permeation following topical administration. In addition, surface functional modification of ethosomes with MAP exerted no effect on vesicle elasticity, which may be attributed to that MAP only coated the vesicle surface without intercalating into the phospholipid bilayer, and the high concentration of propylene glycol in the formulation maintained high fluidity of the lipid membrane.

#### 3.2.5 Short-term stability

**Fig. 2:**
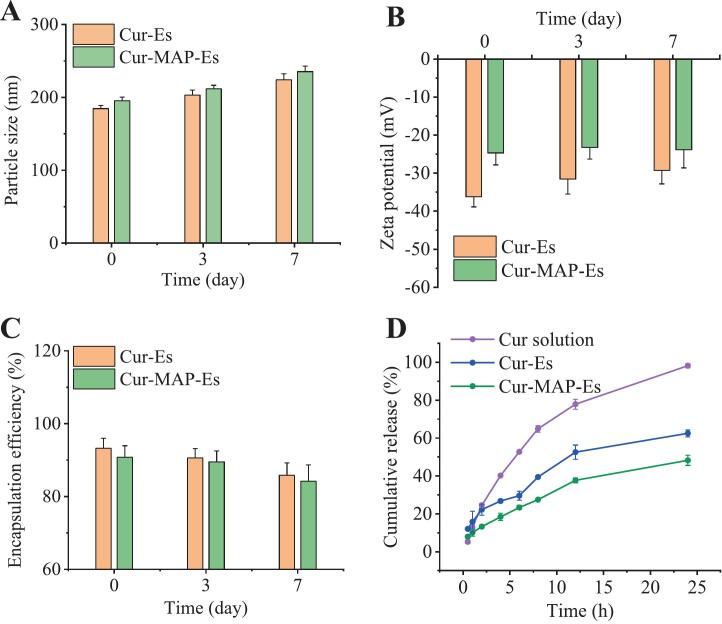
The stability of Cur-Es and Cur-MAP-Es at 4 °C by (A) Partice size, (B) Zeta potential and (C) Encapsulation efficiency. (D) In vitro release profiles of Cur from Cur solution, Cur—Es, and Cur-MAP-Es.

Owing to the impact of environmental factors, leakage and particle aggregation might occur in vesicle samples during storage(de Lange et al., 2020). To assess short-term storage stability, Cur-Es and Cur-MAP-Es were formulated and maintained at 4 °C for a duration of 7 days. An upward trend in particle size was observed for all formulations (Fig. 2A), concurrent with a downward trend in both the absolute zeta potential and EE (Fig. 2B and Fig. 2C). However, no significant changes were noted in these physicochemical parameters when compared to those recorded at day 0, demonstrating the ability of Cur-MAP-Es to maintain good encapsulation performance and uniform dispersion under low temperature conditions over a short storage period.

### 3.3 In vitro drug release

The release curves showed that Cur-Es and Cur-MAP-Es had an initial rapid release of 12.06% and 7.92%, respectively, succeeded by a continuous sustained release phase(Fig. 2D). The percentage of Cur released at 24 h was 98.17%, 62.49% and 42.23% for Cur solution, Cur-Es and Cur-MAP-Es, respectively. The vesicle formulations exhibited a significantly reduced cumulative release of Cur relative to the free form of the drug(*p* < 0.05). The observed initial burst release originates from the fast initial liberation of Cur located close to the vesicle surface, while the lipid bilayer acts as a barrier retarding outward diffusion of encapsulated Cur and thus substantially reducing the drug release rate in the later release phase(Mohapatra et al., 2023). This release profile from the vesicle formulations is therapeutically beneficial for topical skin diseases, wherein the initial burst provides a rapid therapeutic dose, whereas the subsequent sustained release prolongs the pharmacological effect(Wang et al., 2025).

### 3.4 FTIR analysis

**Fig. 3:**
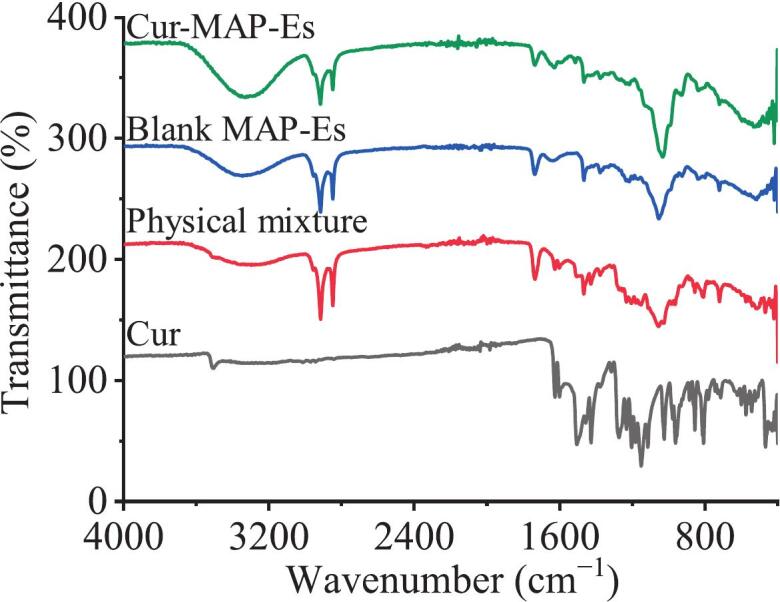
FTIR spectra of Cur powder, the physical mixture of Cur and MAP-Es, blank MAP-Es and Cur-MAP-Es.

The comparative spectra are depicted in Fig. 3. In the pure drug spectrum, the peak at 3507.88 cm^−1^ was assigned to O − H stretching of the phenolic hydroxyl (O − H) groups. The peaks observed at 1625.69 cm^−1^ and 1600.63 cm^−1^ were attributed to the tensile vibration of ketone carbonyl (C=O) groups and bending vibration associated with benzene ring skeletons (C=C), respectively. Additionally, a peak appearing at 962.30 cm^−1^ was associated with the tensile vibration of the olefinic (C − H) groups present on the aromatic ring(Zhang et al., 2024). In comparison with the infrared spectrum of Cur-MAP-Es, most characteristic peaks associated with Cur had diminished or disappeared entirely, indicating that vesicles exerted a shielding effect on Cur. Furthermore, in the spectrum of blank MAP-Es and physical mixture, in both spectra of blank MAP-Es and physical mixtures, the aliphatic O—H tensile vibration peak was identified at 3342.03 cm^−1^, while in the spectrum for Cur-MAP-Es, the intensity of the peak was significantly enhanced, indicating that new hydrogen bonds were formed between Cur and vesicles, thereby contributing to greater structural stability for the vesicular system(Ashin et al., 2024).

### 3.5 In vitro skin permeation and retention

**Fig. 4:**
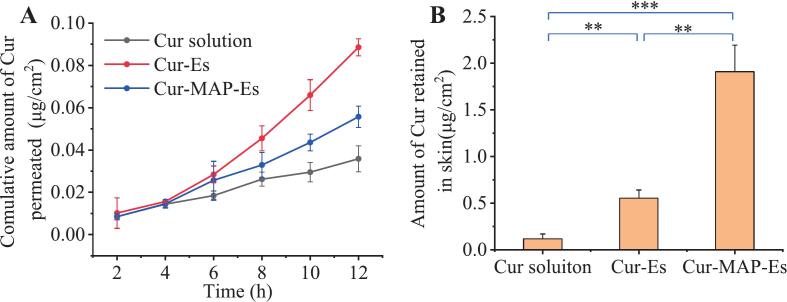
(A) Skin permeation profiles of Cur from Cur solution, Cur—Es, and Cur-MAP-Es. (B) Cur retained in porcine skin following 12 h of exposure to the Cur solution, Cur—Es, and Cur-MAP-Es (**p < 0.01, ***p < 0.001).

Porcine skin displays similar morphological characteristics and barrier functions to human skin, thus representing an optimal animal skin alternative for in vitro skin permeation investigations that simulate in vivo environments(Herbig et al., 2015). The permeation results of the test samples through porcine skin are illustrated in Fig. 4A. Cur-Es and Cur-MAP-Es showed 2.47- and 1.55-fold greater cumulative skin permeation than the Cur solution at 12 h. The enhanced skin penetration achieved by vesicle formulations may stem from the inclusion of propylene glycol acting as a penetration promoter, which might induce reversible alterations in the lipids and structure of the skin(Carrer et al., 2020). Furthermore, phospholipid bilayer vesicles containing propylene glycol have the potential to enhance both ultra-deformability and flexibility of the vesicles, enabling them to pass through the microscopic pores and interstitial spaces within the dermal tissue, thereby improving drug permeability(Kumar et al., 2025). Additionally, the nano-sized dimensions and high lipid content inherent in lipid vesicles contribute to a sealing effect that helps prevent transdermal moisture loss while simultaneously increasing hydration levels within the skin, which promotes more effective drug permeation into cutaneous layers(Rapalli et al., 2023). Notably, comparative analysis of Cur-MAP-Es and Cur-Es showed that Cur-MAP-Es exhibited a significantly lower cumulative skin permeation at 12 h (*p* < 0.05), this phenomenon might result from biological adhesion properties associated with Cur-MAP-Es which impede rapid drug diffusion through the skin. These findings suggested that Cur-MAP-Es could facilitate increased drug permeation across the SC, which would be beneficial to improve the topical therapeutic effects on dermatological conditions.

The amount of drug retained within the skin after the in vitro permeation test was quantified and is presented in Fig. 4B. The retention values for Cur solution, Cur—Es, and Cur-MAP-Es were determined to be 0.12, 0.55, and 1.91 μg/cm^2^, respectively. This represents a substantial enhancement for Cur-MAP-Es, which exhibited 3.47-fold and 15.92-fold greater skin retention than Cur-Es and the Cur solution. Previous studies have shown that the skin drug retention capacity of hyaluronan-modified ethosomes is only approximately three times that of Cur solution (Zhang et al., 2019). Therefore, Cur-MAP-Es were developed as a novel adhesive delivery system, which endows it with excellent skin retention. The enhanced skin retention observed with Cur-MAP-Es might be attributed to the fusion of lipophilic components present in vesicles with the skin, leading to the formation of drug reservoirs that facilitate sustained and slow drug release within the skin(Nasr et al., 2019). Furthermore, the larger particle size of Cur-MAP-Es relative to Cur-Es may represent an additional reason for the improved drug skin retention(Yokota and Kyotani, 2018). Additionally, it is speculated that the features such as positive charge, DOPA groups, and hydrophobic regions in the molecular structure of MAP might interact with skin cells, resulting in enhanced adhesion properties for Cur-MAP-Es and improved drug retention within the skin (Guo et al., 2020). In summary, Cur-MAP-Es demonstrated an ability to increase skin retention while simultaneously reducing permeation of Cur into systemic circulation. These results highlight the unique advantage of the MAP-modified delivery system in achieving targeted skin therapy, which contributes to improved topical efficacy. Furthermore, the improved drug retention in the skin helps to reduce systemic drug exposure, thereby minimizing potential drug-related widespread side effects.

### 3.6 Intradermal fluorescence distribution

**Fig. 5:**
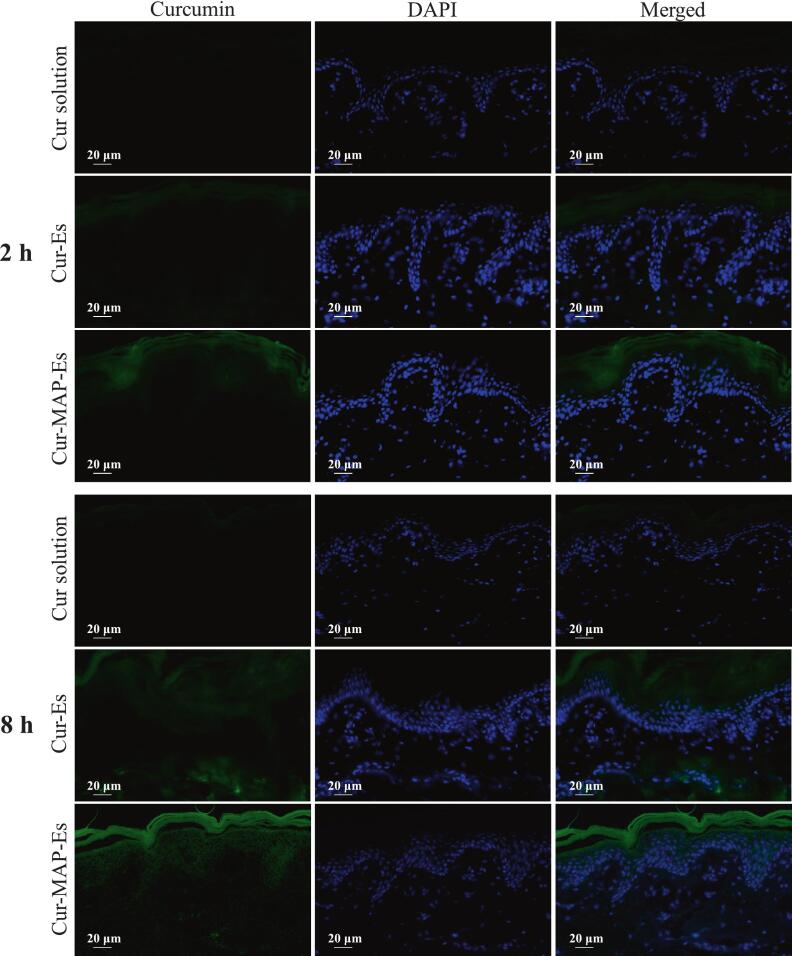
Fluorescence micrographs of porcine skin longitudinal sections following application of Cur solution, Cur—Es, and Cur-MAP-Es.

As illustrated in Fig. 5, the fluorescence intensity and distribution observed after treatment with the Cur formulation revealed that a substantial amount of green fluorescence (representing Cur) was detected above the blue DAPI-stained nuclei at 2 h post-treatment. It is well established that the SC represents the outermost layer of the epidermis, consisting primarily of enucleated dead keratinocytes(Chen et al., 2024). Therefore, this result indicated that most drugs were localized within the SC. Skin samples exposed to the Cur solution showed weak accumulation in the SC, indicating that its permeation into the skin was limited due to an absence of osmotic enhancers or nanocarriers, resulting in almost no fluorescence signal in the dermal region. In contrast, skin treated with Cur-Es and Cur-MAP-Es displayed significantly higher fluorescence intensity in the SC compared with the skin treated with Cur solution, and some green fluorescence coexisted harmoniously with blue fluorescence (nucleus) from the epidermis, indicating effective drug permeation into active epidermal layers, this might be attributable to the super-deformability of these elastic vesicles, which could enable them to navigate the minute intercellular gaps in the SC and reach the viable epidermis(El-Menshawe et al., 2019). Notably, skin specimens exposed to Cur-MAP-Es displayed green fluorescence confined to the epidermis. In contrast, Cur delivered by Cur-Es penetrated into deeper layers, with fluorescence observed in the dermis. After administration for 8 h, it became evident that Cur-Es had effectively permeated and distributed throughout all layers of skin. The fluorescence intensity clearly reflected the efficient delivery of Cur-Es to deeper skin layers and the ability of the preparation to facilitate drug penetration across the SC and into the dermis. However, in the case of Cur-MAP-Es, most of the green fluorescence signal remained localized within the epidermis, with only a small amount was observed in the dermis, indicating that most of the Cur would be effectively retained within the epidermis after being delivered into the skin via vesicles. This could stem from the combined effects of vesicle deformability, particle size, surface charge, and bioadhesion. Therefore, Cur-MAP-Es could be proposed for enhancing drug permeation following percutaneous administration to achieve topical therapeutic benefits.

### 3.7 Interaction mechanisms of Cur-MAP-Es with the skin

#### 3.7.1 HE analysis of skin structure

**Fig. 6:**
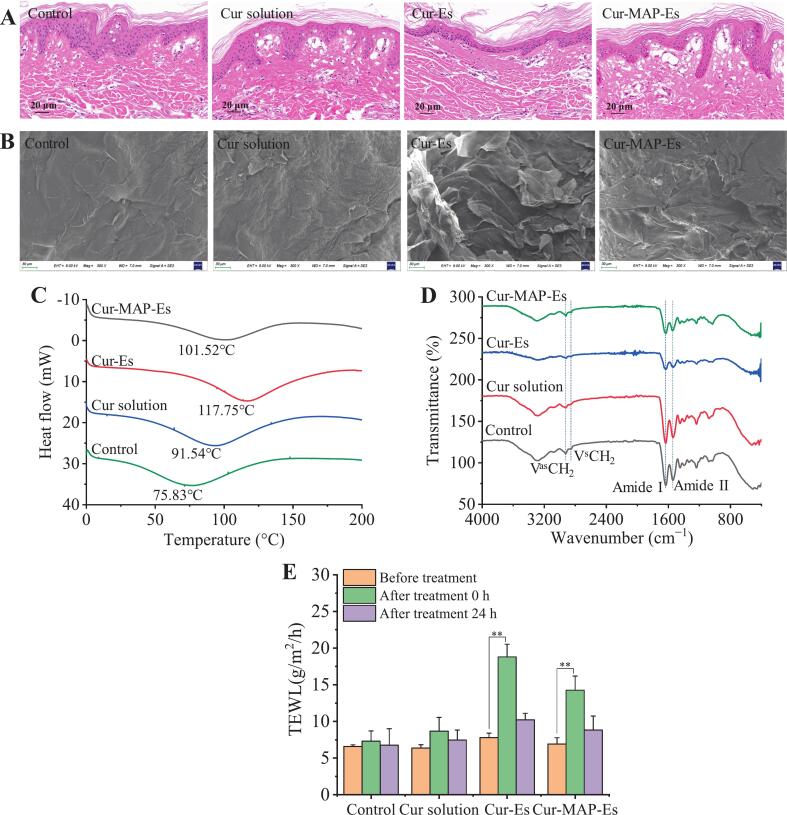
(A) Histological photomicrographs of skin sections. (B) SEM photomicrographs of skin surface. (C) DSC thermograms and (D) FTIR spectra of untreated porcine skin, and skin administered with Cur solution, Cur-Es and Cur-MAP-Es. (E) Effect of the Cur solution, Cur-Es and Cur-MAP-Es on TEWL values (**p < 0.01).

**Fig. 7:**
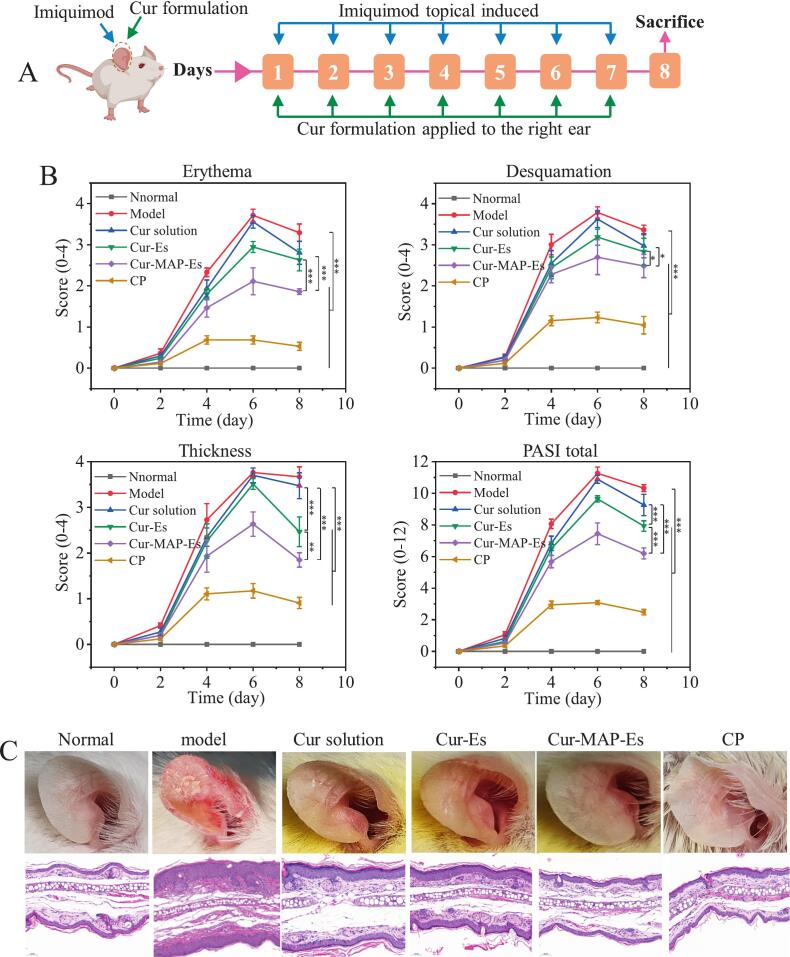
(A) Graphical depiction of the construction protocol for the psoriasis mouse model; (B) PASI scores of auricular skin lesions in mice with IMQ-induced psoriasiform inflammation after intervention with different preparations; (C) Appearance of ear lesions in mice with IMQ-induced psoriasis-like skin lesions following treatment with various formulations and histopathological examination results of ear tissues in each group on day 8 (*p < 0.05, **p < 0.01, ***p < 0.001).

It could be seen from Figs. 6A that the normal skin without any treatment and the skin treated with Cur solution exhibited a complete structure with each layer arranged closely and distinctly. The outermost layer of the SC displayed a ribbon-like structure that was tightly connected to the epidermis, and the SC did not exhibit flaking or dissociation, thereby providing an effective barrier for skin permeation. In contrast, after treatment with Cur—Es, the layered architecture of the skin appeared disordered, the basal layer was not arranged clearly, and the SC was obviously loose and appeared to fall off. These changes could arise from the migration of ultra-flexible ethosomes that increase the interlayer spacing inside the SC lipid bilayer (Mohapatra et al., 2023). Furthermore, propylene glycol, a major component of ethosomes, acts as an osmotic enhancer that promotes skin permeation of the drug and is postulated to affect the lipid bilayer structure of the skin, thereby affecting the intercellular space of the SC(Sudhakar et al., 2021). Skin treated with Cur-MAP-Es demonstrated swelling in the SC along with a looser connection to the epidermis, but the exfoliation observed was less pronounced compared to that seen in Cur-Es treated skin (Figs. 7D). This difference might be attributable to the beneficial effects of MAP on skincare(Wu et al., 2024). Therefore, by examining changes in skin ultrastructure, it could be inferred that that the role of Cur-Es and Cur-MAP-Es in promoting drug skin permeation might be related to the change of skin structure.

#### 3.7.2 SEM of skin surface

Consistent with the HE findings, the SEM images revealed an intact surface morphology in the untreated skin, and a similar morphological structure was likewise observed in the Cur solution-treated skin (Fig. 6B). This observation was anticipated, as there were no nanocarriers or permeation enhancers present. In contrast, following exposure to the vesicle formulations of Cur-Es and Cur-MAP-Es, the skin surface displayed looseness and cracks, and a considerable detachment of SC fragments from the skin. These vesicular formulations appeared to disrupt the dense architecture of SC cells, thereby facilitating drug penetration. Notably, compared to the Cur—Es, the exfoliation of SC was less pronounced after treatment with Cur-MAP-Es, which might be attributed to skin barrier repair properties of MAP(Wu et al., 2024), suggesting that Cur-MAP-Es could reduce the skin irritation associated with the formulation.

#### 3.7.3 DSC analysis of skin thermal behavior

The DSC spectra of skin treated with different formulations are presented in Fig. 6C. Untreated skin exhibits a characteristic endothermic peak at 75.83 °C, which corresponds to the melting phase transition of intercellular lipids in the SC(Li et al., 2019). After treatment with Cur solution, Cur—Es, and Cur-MAP-Es, the lipid-associated endothermic peaks disappeared, demonstrating that all three formulations disrupted the ordered structure of SC lipids. Meanwhile, skin samples treated with Cur solution, Cur—Es, and Cur-MAP-Es exhibit endothermic denaturation peaks at 91.54 °C, 117.75 °C, and 101.52 °C, respectively. These peaks are attributed to keratin denaturation(Li et al., 2019), confirming that the formulations interact with not only SC lipids but also keratin. The enthalpy changes of keratin denaturation for the Cur solution, Cur—Es, and Cur-MAP-Es treatment groups are 4.86 J/g, 4.98 J/g, and 1.35 J/g, respectively. Notably, the Cur-Es group shows the highest denaturation temperature and the greatest enthalpy change, suggesting an interaction between Cur-Es and keratin that enhances the thermal stability of keratin(Goh et al., 2022). By contrast, the skin treated with Cur-MAP-Es presents a moderate denaturation temperature and the lowest enthalpy change. This phenomenon may be explained by the adhesion of MAP to the keratin(Li and Cao, 2019), which may alter the microenvironment of keratin or form cross-linked structures with keratin, thereby modulating the thermal phase transition behavior of keratin.

#### 3.7.4 FTIR analysis of skin lipid and keratin

FTIR could be used as a valuable tool for assessing the impact of formulation on the SC by comparing the characteristic infrared absorption peaks of skin treated with formulations to the untreated skin. These peaks are associated with the molecular vibrations of SC components, including ceramides, cholesterol, fatty acids, and keratin. As displayed in Fig. 6D, untreated skin displayed characteristic peaks at 2921.63 cm^−1^ and 2853.94 cm^−1^, ascribed to the asymmetric and symmetric C—H stretching vibrations, which are mainly associated with skin lipids. Additionally, the spectrum also exhibited characteristic amide vibrations of keratin, with the C=O stretch (amide I) at 1633.41 cm^−1^ and the C—N stretch (amide II) at 1546.63 cm^−1^.The intensity variations of these characteristic peaks could reflect the alterations in lipid and protein structures of the SC(Gao et al., 2023). In comparison to untreated skin samples, there were no obvious variations were observed in the characteristic absorption peaks of SC treated with Cur solution. However, after treatment with Cur-Es or Cur-MAP-Es, a decrease in peak intensity for both lipids and keratin was noted to some extent, indicating that the vesicle formulation may induce lipid disorganization and keratin denaturation within skin tissue, which provided favorable conditions for the rapid permeation and absorption of Cur into skin tissue. In addition, we observed that after treatment with Cur—Es, the peak intensities of skin lipids and keratin decreased to a greater extent compared with Cur-MAP-Es. This may be attributed to the fact that MAP protein possesses skin-protective or barrier-repairing properties(Luo et al., 2024), thereby alleviating the direct impact of ethosomes on the structure of skin lipids and keratin.

#### 3.7.5 TEWL measurements

Impaired skin barrier function will lead to increased skin water loss(Montero-Vilchez et al., 2021). The TEWL value of the skin did not change much before and after treatment with physiological saline or Cur solution, whereas a significant rise was observed following application of Cur-Es or Cur-MAP-Es for 12 h (Fig. 6E), demonstrating that the structural modifications of lipids and keratin caused by the vesicle system undermined the skin barrier. The highest TEWL value was related to Cur—Es, which indicated that propylene glycol mediated the maximum interaction between the vesicles and SC, while Cur-MAP-Es reduced the TEWL value compared with the skin treated with Cur-Es due to the moisturizing effect of MAP molecules on the skin surface. Nevertheless, TEWL levels returned to baseline within 24 h post-treatment, which indicated a temporary impact of these ethosomes(Wang et al., 2021).

### 3.8 In vivo anti-psoriatic effect

Throughout the entire experimental period, the mice assigned to the normal group maintained their food consumption and activity levels within normal ranges. In contrast, mice treated with IMQ progressively developed behavioral deficits, including listlessness, a curled posture, and reduced food intake. In addition, IMQ also induced dermatological symptoms such as skin inflammation and lackluster fur. The application of Cur formulations and CP cream notably ameliorated these induced behavioral deficits and led to the recovery of food consumption.

The administration scheme is shown in Fig. 7A. In contrast to the normal group, the mice in the model group presented with slight erythema on their ears following 1 day of IMQ administration. Obvious desquamation appeared on the ears at 3–4 days, while the extent of ear injury hit its peak in the 5–6 days period. A slight alleviation of symptoms was detected following 7 days of IMQ administration. The Cur formulations exhibited the capacity to suppress inflammation within mouse ear tissue, but the efficacy was inferior compared to that of CP cream. Correspondingly, the scores for erythema, desquamation, ear thickening, and PASI in IMQ-treated group reached their peak values on day 6, while a significant reduction in PASI scores was observed in the Cur formulation and CP groups, demonstrating their therapeutic efficacy against psoriatic lesions. Importantly, Cur-MAP-Es demonstrated superior performance among the tested formulations, achieving the most pronounced inhibition of psoriatic symptoms. On day 6, the PASI score in the Cur-MAP-Es group was only 0.68 and 0.77 times relative to the Cur solution and Cur-Es groups, respectively. (Fig. 7B). Upon reaching the eighth day, a significant reduction in erythema, desquamation, thickening, and PASI scores was observed in all treated groups as compared with the model group (*p* < 0.05). Specifically, the PASI scores of the Cur-MAP-Es group and the Cur-Es group were found to be 0.67 times and 0.86 times that of the Cur solution group, respectively, and Cur-MAP-Es proved to be the most effective among all evaluated Cur formulations.

At the conclusion of the experiment on Day 8, the recovery status of inflammatory symptoms in the auricular skin of mice is illustrated in Fig. 8C. Mice in the model group presented with severe auricular skin lesions, which were characterized by prominent scale formation, skin thickening, and marked erythema within the lesioned regions. In contrast, auricular inflammation was alleviated to varying degrees in mice across all drug-administered groups. Notably, the Cur-MAP-Es group exhibited the most pronounced improvement, with the auricles of mice in this group appearing smoother than those in other drug-administered groups and showing no obvious wrinkling.

Skin histopathological alterations in mice from each experimental group were evaluated, as shown in Fig. 7C. The auricular skin tissue of mice in the normal group remained structurally intact, with no significant histopathological abnormalities observed. In contrast, psoriatic model mice exhibited characteristic psoriasis-associated pathological alterations in the skin, including extensive proliferation of epidermal spinous cells, acanthosis, extensive coverage of the skin surface by a thick layer of parakeratotic tissue, downward extension of epidermal pegs in a club-like morphology, and massive infiltration of inflammatory cells. Compared with the model group, mice in each treatment group showed varying degrees of improvement in skin histopathological damage. Among all treatment groups, the Cur-MAP-Es group achieved the optimal recovery of skin histopathological morphology, with the degree of improvement achieved was on par with the positive control. This histopathological finding was consistent with both the gross observations of mouse skin appearance and the results of skin inflammation scoring. These data collectively suggest that Cur-MAP-Es can fully exert its pharmacological activity by improving the cutaneous permeation and retention of the drug, thereby exerting a more superior therapeutic effect on the IMQ-induced mouse model of psoriasis.

## 4 Conclusions

In the present work, we developed MAP-modified curcumin-loaded ethosomes to enhance skin permeation and accumulation of Cur, thereby achieving effective psoriasis treatment. The formulated Cur-MAP-Es exhibited spherical morphology with excellent elasticity and encapsulation efficiency for Cur. Our findings indicated that Cur-MAP-Es provided a sustained release of Cur and markedly improved drug retention within the skin versus the unmodified Cur—Es. A substantial portion of the Cur in Cur-MAP-Es could be effectively retained in epidermis after being delivered into the skin. The Cur-MAP-Es facilitated enhanced skin absorption of the drug via reversible alterations in the structure of the SC through lipid distortion and keratin denaturation. Moreover, topical application of Cur-MAP-Es could significantly alleviate the inflammatory symptoms of IMQ-induced psoriatic dermatitis in mice. Therefore, Cur-MAP-Es might provide a promising formulation for topical percutaneous delivery of Cur.

## CRediT authorship contribution statement

**Jiangxiu Niu:** Writing – original draft, Methodology, Formal analysis, Data curation, Conceptualization. **Ming Yuan:** Visualization, Investigation, Data curation, Conceptualization. **Liye Wang:** Software, Conceptualization. **Pei Zhang:** Methodology, Investigation, Data curation. **Jucai Wang:** Software, Data curation. **Xianming Liu:** Validation, Data curation.

## Ethical approval

All animal experiments complied with institutional and national guidelines, with approval granted by the Institutional Animal Care and Use Committee of Luoyang Normal University (Approval No. LYNUIACUA–2025–06.142–058).

## Funding

This study was supported by the 10.13039/501100001809National Natural Science Foundation of China (32101150), the Key Scientific and Technological Project of Henan Province of China (262102311208 and 242102110051), the Training Program for Young Backbone Teachers in Higher Education Institutions of Henan Province (2025GGJS110).

## Declaration of competing interest

The authors declare no conflicts of interest relevant to this study.

## Data Availability

All data generated or analyzed during this study are included in this published article.
